# Genome-Wide Identification of a Chromatin-Splicing Regulatory Axis Driven by DOT1L in MLL-Rearranged Acute Myeloid Leukemia

**DOI:** 10.3390/genes17080857

**Published:** 2026-07-24

**Authors:** Qi Liu, Bowen Yang, Tianbao Li, Raven Garcia, Shengtong Han, Victor X. Jin

**Affiliations:** 1 Department of Bioinformatics, Drylab Inc., Quincy, MA 02171, USA; 2 Department of Diagnostic and Interventional Imaging, University of Texas Health Science Center at Houston, Houston, TX 77030, USA; bowen.yang@uth.tmc.edu; 3 Department of Molecular Medicine, UT Health San Antonio, San Antonio, TX 78229, USA; 4 School of Dentistry, Marquette University, Milwaukee, WI 53233, USA; 5 Data Science Institute, Medical College of Wisconsin, Milwaukee, WI 53226, USA

**Keywords:** acute myeloid leukemia, DOT1L, H3K79 methylation, alternative splicing, exon skipping, MLL rearrangement, epigenetic regulation, RNA processing

## Abstract

**Background/Objectives:** Aberrant H3K79 dimethylation (H3K79me2) by DOT1L is a defining feature of MLL-rearranged (MLLr) acute myeloid leukemia (AML), but whether this modification influences alternative splicing is unclear. We examined the relationship between H3K79me2 and exon skipping in primary MLLr AML. **Methods:** We performed H3K79me2 ChIP-seq and RNA-seq on primary samples from 24 MLLr AML patients, 4 wild-type MLL AML patients, and 4 healthy bone marrow donors, with matched profiling before and after DOT1L inhibition using EPZ5676. DOT1L co-immunoprecipitation with mass spectrometry (IP-MS) was performed in MV-4-11 and MOLM-14 cells, and an aggregate splicing score was evaluated in the TCGA-AML cohort. **Results:** A subset of exon skipping (SE) events was enriched at H3K79me2-occupied loci in MLLr samples. EPZ5676 remodeled SE patterns, and many switched events showed concurrent loss of local H3K79me2. Genes harboring these events were enriched for RNA processing, DNA repair, and apoptosis functions. Core spliceosomal and hnRNP proteins were prominent in the DOT1L interactome and were reduced after EPZ5676, and SRSF2 and hnRNPA1 binding motifs were enriched near the regulated exons. About two-thirds of the common switched events overlapped a local H3K79me2 peak, and an aggregate splicing score derived from these events stratified patients by overall survival in the TCGA-AML cohort, with higher scores associated with shorter survival. **Conclusions:** These data support a model in which H3K79me2 contributes to alternative splicing regulation in MLLr AML, linking the core epigenetic lesion of this disease to aberrant RNA processing with potential prognostic relevance.

## 1. Introduction

Acute myeloid leukemia (AML) with rearrangements of the mixed lineage leukemia gene (MLL/KMT2A) is an aggressive disease subtype associated with poor outcomes [1,2,3]. These rearrangements account for 5–10% of adult and 15–20% of pediatric AML cases, with high relapse rates and inferior survival relative to other cytogenetic groups [2,4]. The MLL gene encodes a histone H3 lysine 4 (H3K4) methyltransferase that plays essential roles in hematopoietic development and gene regulation. Chromosomal translocations fuse the N-terminal portion of MLL to various partner genes, generating oncogenic fusion proteins that drive leukemogenesis through aberrant transcriptional activation of HOXA cluster genes and MEIS1 [5].

MLL-rearranged (MLLr) leukemia is characterized by aberrant recruitment of DOT1L, the only H3K79 methyltransferase, to MLL-fusion target genes [6,7,8]. MLL fusion partners such as AF4, AF9, AF10, and ENL interact with DOT1L or its associated complex, resulting in H3K79 hypermethylation at target loci and sustained pro-leukemic gene expression [9]. Both genetic and pharmacological studies confirm that DOT1L activity is essential for MLLr leukemia initiation and maintenance [10,11,12]. The DOT1L inhibitor EPZ5676 (pinometostat) has shown selective activity against MLLr leukemia cells in preclinical models and has been evaluated in clinical trials [13]. DOT1L has also been implicated as a therapeutic target in DNMT3A-mutant AML, which points to broader relevance beyond MLLr disease [14].

Beyond transcriptional control, histone modifications also appear to regulate RNA processing, particularly alternative splicing [15,16,17]. Alternative splicing expands proteome diversity and its dysregulation contributes to cancer pathogenesis [18]. Two models explain chromatin effects on splicing: kinetic coupling, where histone modifications alter RNA polymerase II elongation and splice site recognition, and chromatin-adaptor recruitment, where histone marks directly recruit splicing factors [19,20,21]. Genome-wide analyses show non-random histone modification patterns around alternatively spliced exons, a finding consistent with functional links between chromatin and splicing [22].

Splicing factor mutations occur frequently in hematologic malignancies and contribute to disease pathogenesis [23,24]. SRSF2, SF3B1, and U2AF1 mutations are recurrent in MDS and AML, causing widespread splicing changes that promote leukemic transformation [25]. SR proteins generally promote exon inclusion while hnRNP proteins favor exon skipping [26,27,28]. Whether splicing regulators intersect with DOT1L-mediated H3K79 methylation in MLLr AML has not been examined.

Here we examined the relationship between H3K79me2 and alternative splicing in primary MLLr AML samples and cell lines. By integrating ChIP-seq of H3K79me2 with RNA-seq, we identified exon skipping events associated with H3K79me2 loci that change upon DOT1L inhibition. Pathway analysis of genes harboring H3K79me2-linked splicing events revealed enrichment for RNA processing, genome maintenance, and apoptosis-related functions. These findings provide an insight into a functional link between DOT1L-mediated chromatin modification and alternative splicing in MLLr AML.

## 2. Materials and Methods

Patient samples and cell lines

Primary bone marrow samples with deidentified patient information were procured from UT MD Anderson Cancer Center, including 24 patients with MLLr acute myeloid leukemia (AML) and four patients with wild-type MLL (wtMLL) AML under UTHSA and MCW approved institutional review board (IRB). Four bone marrow samples of healthy donors were purchased from National Disease Research Interchange (NDRI, Philadelphia, PA, USA). MLL rearrangement status was determined via fluorescence in situ hybridization (FISH) and/or reverse transcription PCR (RT-PCR). A summary of all primary patient and donor samples is provided in Supplementary Table S1. Fresh-frozen samples were used for both ChIP-seq and RNA-seq analyses. Informed consent for participation was obtained by the biobank at the time of sample collection. This study was conducted in accordance with the Declaration of Helsinki.

Human AML cell lines MV-4-11 (MLL-AF4), MOLM-14 (MLL-AF9), and AML-193, as well as the lymphoblastoid cell line GM12878, were obtained from ATCC (Manassas, VA, USA) or DSMZ (Braunschweig, Germany). Cells were maintained in RPMI-1640 medium supplemented with 10% fetal bovine serum and 1% penicillin–streptomycin at 37 °C in a humidified incubator with 5% CO_2_.

ChIP-seq library preparation and sequencing

ChIP-seq profiling was followed with our previous publications [29]. Briefly, primary tissue samples were cross-linked with 1% formaldehyde for 10 min at room temperature, and the reaction was quenched with 125 mM glycine. Nuclei were isolated, and chromatin was sheared to an average fragment size of 200–500 bp using a Covaris E220 sonicator. Immunoprecipitation was performed overnight at 4 °C with an anti-H3K79me2 antibody (Abcam, Waltham, MA, USA, ab3594). Following immunoprecipitation, DNA was purified using the MinElute PCR Purification Kit (Qiagen, Hilden, Germany). Sequencing libraries were prepared using the NEBNext Ultra II DNA Library Prep Kit (New England Biolabs, Ipswich, MA, USA) and sequenced on an Illumina NextSeq 3000 platform with paired-end and single-end reads. Each primary sample was processed as one biological replicate, so that group-level comparisons were based on 24 MLLr AML, 4 wtMLL AML, and 4 healthy-donor samples. For the DOT1L inhibition experiment, untreated and EPZ5676-treated ChIP-seq libraries were prepared from paired aliquots of the same primary MLLr samples.

RNA-seq library preparation and sequencing

Total RNA was extracted using TRIzol reagent (Invitrogen, Waltham, MA, USA) followed by DNase I treatment. RNA integrity was assessed using an Agilent, Santa Clara, CA, USA, 2100 Bioanalyzer, and only samples with an RNA integrity number (RIN) ≥ 7 were used for subsequent analyses. Poly(A)+ RNA was enriched using oligo(dT) magnetic beads. Strand-specific RNA-seq libraries were prepared using the TruSeq Stranded mRNA Library Prep Kit (Illumina, San Diego, CA, USA) and sequenced on an Illumina NextSeq 3000 platform, yielding approximately 50 million reads per sample. RNA-seq was performed on the same set of primary samples, with one library per sample, so that differential splicing was evaluated at the group level across the 24 MLLr AML, 4 wtMLL AML, and 4 healthy-donor samples. For the cell-line IP-MS experiment described below, three biological replicates per condition were used.

DOT1L co-immunoprecipitation and mass spectrometry (IP-MS)

MV-4-11 (MLL-AF4) and MOLM-14 (MLL-AF9) cells were treated with 2 μM EPZ5676 or DMSO vehicle for 7 days. Approximately 2 × 10^8^ cells per condition were harvested and lysed in IP lysis buffer (50 mM Tris-HCl pH 7.5, 150 mM NaCl, 1 mM EDTA, 0.5% NP-40) with protease and phosphatase inhibitor cocktails (Roche, Basel, Switzerland). Lysates were pre-cleared with Protein A/G magnetic beads and incubated overnight at 4 °C with an anti-DOT1L antibody (Cell Signaling Technology, Danvers, MA, USA, #77087) or matched IgG isotype control. Immunocomplexes were captured on Protein A/G magnetic beads, washed four times with IP lysis buffer, and eluted in SDS sample buffer. Eluates were run on a short-migration SDS-PAGE gel; the stacked protein band was excised and subjected to in-gel trypsin digestion. Tryptic peptides were desalted on C18 StageTips and analyzed by nanoLC–MS/MS on a Thermo, Waltham, MA, USA, Q Exactive HF-X coupled to an EASY-nLC 1200 system, using a 90 min gradient from 5% to 35% acetonitrile in 0.1% formic acid. MS data were acquired in data-dependent top-20 mode. Raw files were searched against the UniProt human database (canonical + isoforms, 2023-10 release) in MaxQuant (v2.4) with label-free quantification, peptide-level FDR of 1%, and match-between-runs across replicates. Proteins were retained when identified by ≥2 unique peptides in at least two of three biological replicates per condition. Interactors were called “vanished” (detected in DMSO but absent after EPZ5676), “decreased” (log_2_FC ≤ −1 and FDR-adjusted *p* < 0.05), “gained” (log_2_FC ≥ 1 and FDR-adjusted *p* < 0.05), or “no change” otherwise. Protein–protein interaction networks were built with STRING (v12, minimum confidence score 0.7) and visualized in Cytoscape (v3.10). GO biological process enrichment on decreased/vanished interactors was performed with Metascape using the whole proteome as background.

Bioinformatics analysis

ChIP-seq reads were aligned to the human reference genome (hg19) using Bowtie2. Peak calling was performed with MACS2 [30] using default parameters with a q-value cutoff of 0.05. Peaks with fold enrichment > 2 were retained, and only peaks reproducibly detected across biological replicates within the same sample group were included in downstream analyses. RNA-seq reads were aligned to hg19 using STAR [31]. Alternative splicing events were identified with rMATS [32], and exon skipping (SE) events were quantified using percent spliced in (PSI) values. Significant SE events were defined by |ΔPSI| > 0.1 and a false discovery rate (FDR) < 0.05. For robustness, SE events had to be detected in at least three samples within a given group. SE events were classified as Unchanged, Switched, or Mixture based on the directionality and consistency of PSI changes across samples. ChIP-seq and RNA-seq data were integrated by assigning H3K79me2 peaks to SE events when peaks overlapped exon bodies or fell within 500 bp of exon–intron boundaries. This approach captured local chromatin modification changes that accompanied splicing regulation. Common SE events across patient samples were identified using an incremental threshold approach.

Functional enrichment analysis

Gene Ontology (GO) enrichment analysis was performed using DAVID [33] and Metascape. Enrichment of KEGG and WikiPathways were assessed to identify associated signaling pathways. RNA-binding protein (RBP) motif enrichment within regulated exon regions was analyzed using RBPmap [34] to predict candidate splicing factor binding sites. To compute the H3K79me2-SE splicing score, PSI values for the 42 common Switched SE events were Z-score normalized and averaged per patient to generate a composite splicing dysregulation index. Survival analyses were conducted on the publicly available TCGA-AML cohort (*n* = 160) [35] using Kaplan–Meier estimates with log-rank tests. Univariate Cox proportional hazards regression was used to estimate hazard ratios for individual SE-associated genes, and multivariate Cox models adjusted for age, WBC, ELN risk, FLT3-ITD status, and NPM1 mutation status. Receiver operating characteristic (ROC) analysis evaluated the prognostic value of the splicing score alone, ELN risk classification alone, and the combined model for 2-year overall survival prediction. 

Statistical analysis

Differential splicing analyses were performed using the statistical framework implemented in rMATS. Multiple testing correction was applied using the Benjamini–Hochberg false discovery rate (FDR) method for all genome-wide comparisons. Wilcoxon rank-sum tests were used for group comparisons of PSI values and splicing scores. Cox proportional hazards models were used for survival analyses. Statistical analyses were performed using R (version 4.0), and a *p* value < 0.05 (or FDR < 0.05 for genome-wide tests) was considered statistically significant.

## 3. Results

### 3.1. H3k79me2 Enrichment in a Subset of Exon Skipping Events in Mllr Aml

**Figure 1:**
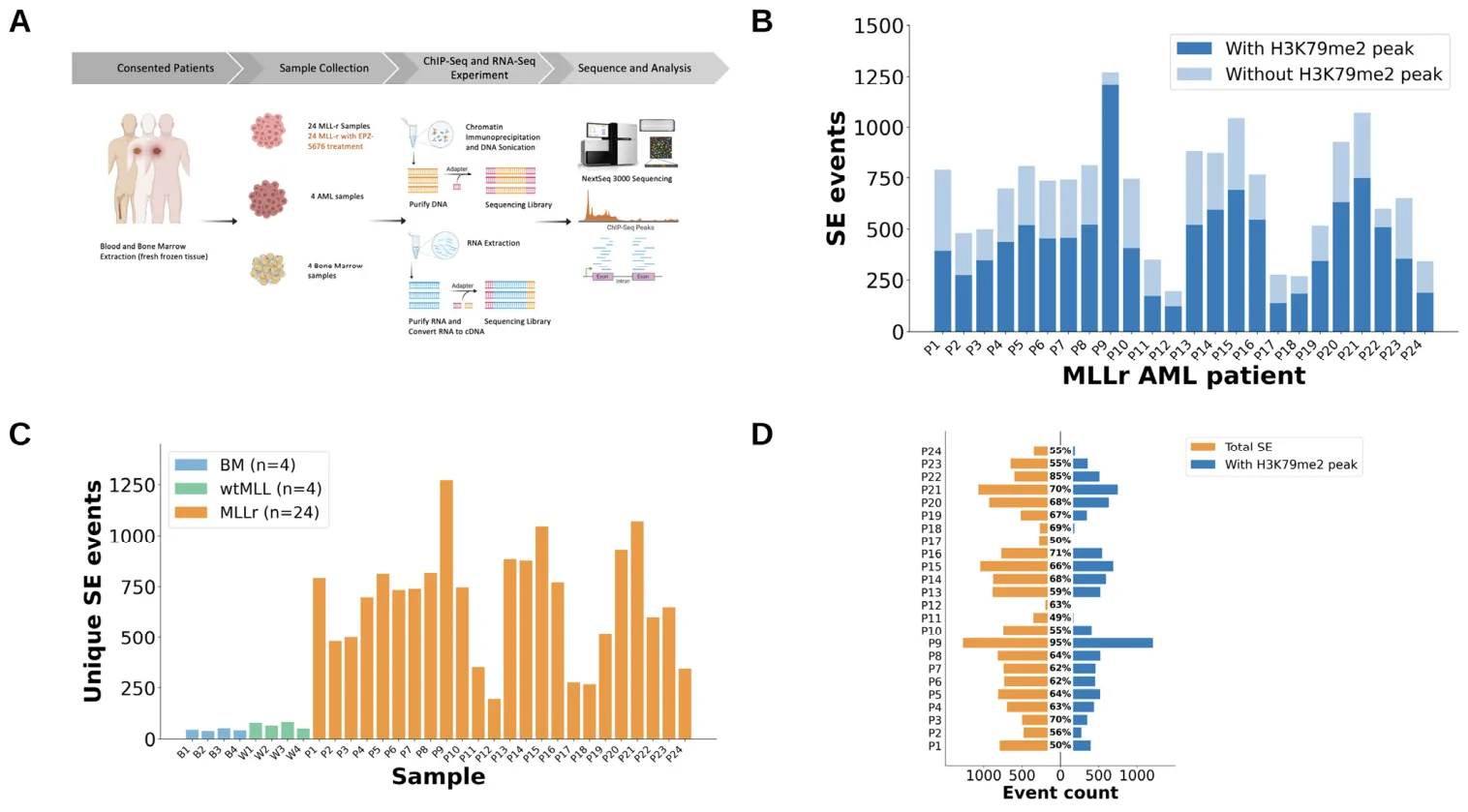
H3K79me2 enrichment is associated with exon skipping events in MLLr AML. (**A**) Schematic overview of the study design. Primary bone marrow samples from 24 MLLr AML patients, 4 wtMLL AML patients, and 4 healthy donors were subjected to H3K79me2 ChIP-seq and RNA-seq to identify H3K79me2-associated exon skipping (SE) events. (**B**) Bar plot showing the number of SE events per MLLr AML sample and the fraction overlapping H3K79me2 peaks. Each bar represents an individual patient sample; colors indicate the proportion of SE events with (dark) or without (light) H3K79me2 peak overlap. (**C**) Bar chart showing the total number of unique SE events (Uni_SE) per sample across BM, wtMLL AML, and MLLr AML groups. MLLr AML samples exhibit substantially higher SE event counts than wtMLL or healthy controls, consistent with aberrant splicing regulation. (**D**) Butterfly bar chart displaying the overlap between Uni SE events (orange, left) and H3K79me2 peaks (blue, right) for each MLLr AML patient. Percentages indicate the proportion of SE events co-localizing with H3K79me2-enriched regions per sample.

We performed an integrated ChIP-seq and RNA-seq analysis on 24 MLLr AML patients, 4 wtMLL AML patients, and 4 healthy bone marrow donors (Figure 1A). ChIP-seq of H3K79me2 showed pronounced enrichment across gene bodies in MLLr compared with wtMLL and healthy controls, consistent with aberrant DOT1L recruitment (Supplementary Figure S1). Across all 24 MLLr samples, we identified a range of 196–1272 unique exon skipping (SE) events per sample, and 49.3–95.1% of these overlapped H3K79me2 peaks (Figure 1B). SE events were classified as Unchanged, Switched, or Mixture based on PSI value consistency. Switched SE events, which represent coordinated splicing changes, were significantly more frequent in MLLr than in wtMLL or healthy samples (Figure 1C). Most of these Switched SE events co-localized with H3K79me2-enriched regions in exon bodies or flanking introns (Figure 1D). This co-localization is compatible with a functional relationship between H3K79me2 deposition and splicing regulation in MLLr. Other splicing event types (A5SS, A3SS, MXE, RI) were also more frequent in MLLr samples, but exon skipping showed the largest fold increase relative to controls (Supplementary Figure S2).

### 3.2. Dot1l Inhibition Induces Coordinated Remodeling of Exon Skipping Patterns Accompanied by Local H3k79me2 Loss

**Figure 2:**
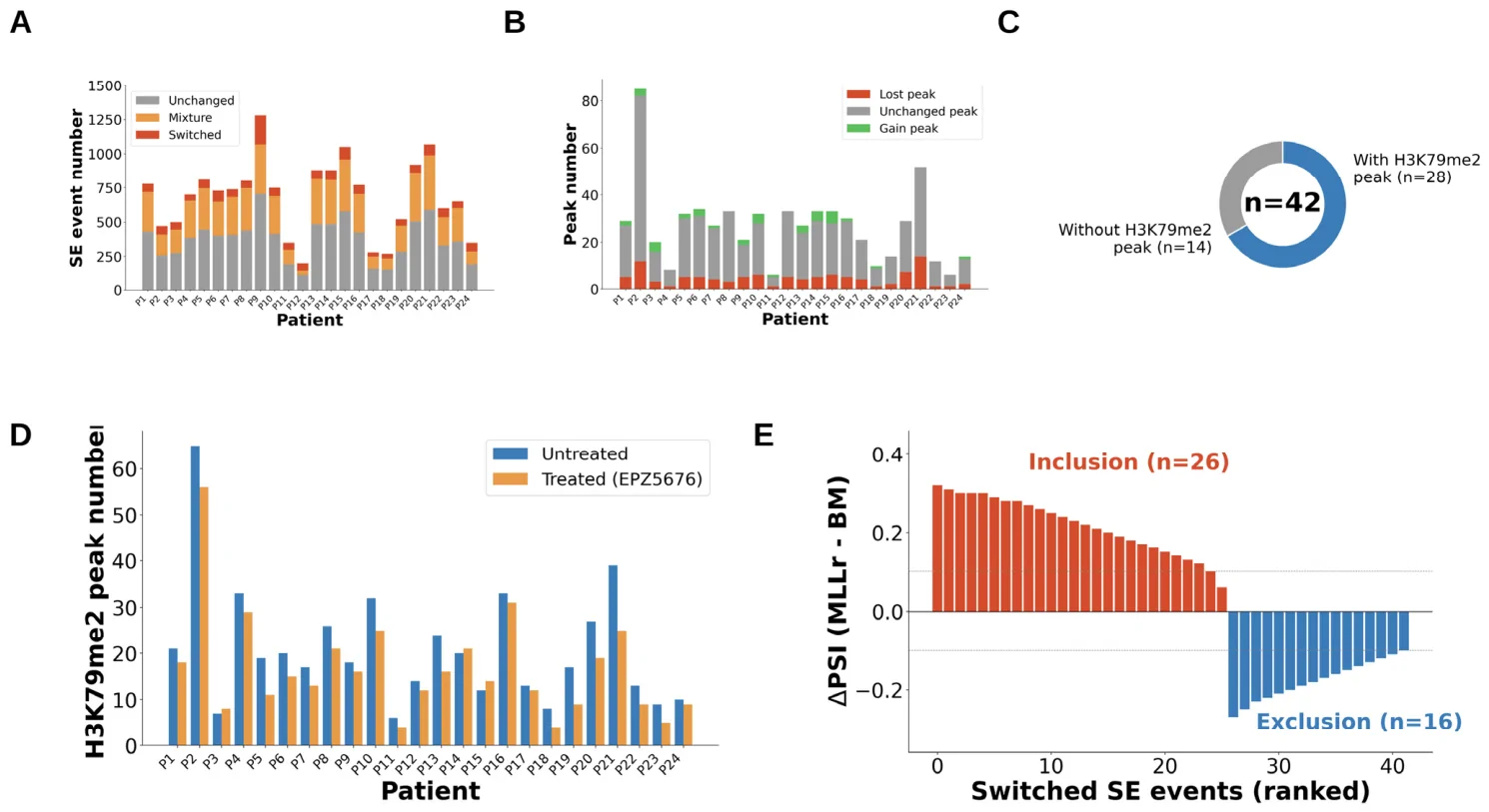
DOT1L inhibition induces coordinated remodeling of exon skipping patterns with concurrent H3K79me2 loss. (**A**) Stacked bar chart of the number of SE events per MLLr AML patient sample, classified as Unchanged, Mixture, or Switched. (**B**) Stacked bar chart of H3K79me2 peak changes (Lost Peak, Unchanged Peak, Gain Peak) per patient following EPZ5676 treatment. (**C**) Donut chart of the proportion of the 42 common Switched SE events that co-localize with H3K79me2 peaks (*n* = 28, 66.7%) versus those without peak overlap (*n* = 14, 33.3%). (**D**) Grouped bar chart comparing the total number of H3K79me2 peaks per patient before (untreated) and after EPZ5676 treatment (treated); DOT1L inhibition produced a global reduction in H3K79me2 peaks (*p* < 0.01, paired *t*-test). (**E**) Waterfall plot of ΔPSI values for the 42 common Switched SE events ranked by magnitude of change. Red bars, exon inclusion events; blue bars, exon exclusion events.

To test whether H3K79me2 functionally regulates splicing, we treated MLLr AML samples with the DOT1L inhibitor EPZ5676 and performed paired ChIP-seq and RNA-seq analyses. EPZ5676 treatment induced widespread changes in SE patterns. Using an incremental threshold approach, we identified Switched SE events shared across multiple patient samples: 344 events appeared in at least one sample, with progressively fewer events shared across 2 (105), 3 (67), and more samples (Figure 2A; Supplementary Figure S5). Among these, 80 Switched SE events showed concurrent H3K79me2 peak loss, with 23, 14, and 9 events shared across 2, 3, and 4 samples respectively (Figure 2B). Of the 42 common Switched SE events, 28 (66.7%) co-localized with H3K79me2 peaks, a strong chromatin-splicing association (Figure 2C). Quantitative analysis confirmed that total H3K79me2 peak number decreased globally following DOT1L inhibition across all patient samples (Figure 2D). Waterfall analysis of the 42 common Switched SE events showed a mixture of exon inclusion and exclusion changes, and most events showed increased inclusion in MLLr compared with BM controls (Figure 2E). On this basis, of the 42 common Switched events, 28 (about two-thirds) overlapped a local H3K79me2 peak and are the candidates for direct, locally coupled regulation, whereas the remaining 14 (about one-third) lacked local peak overlap and are more likely to reflect indirect or transcription-coupled consequences of DOT1L inhibition. This split should be read as an approximate upper bound on the directly coupled fraction, because peak overlap indicates proximity rather than a defined regulatory mechanism. Representative genome-browser tracks of H3K79me2 occupancy at SRSF2, BCL2L1, and FLT3 illustrate this local reduction after EPZ5676 treatment (Supplementary Figure S8).

### 3.3. Dot1l-Regulated Splicing Events Are Enriched in RNA Processing and Genome Maintenance Pathways

**Figure 3:**
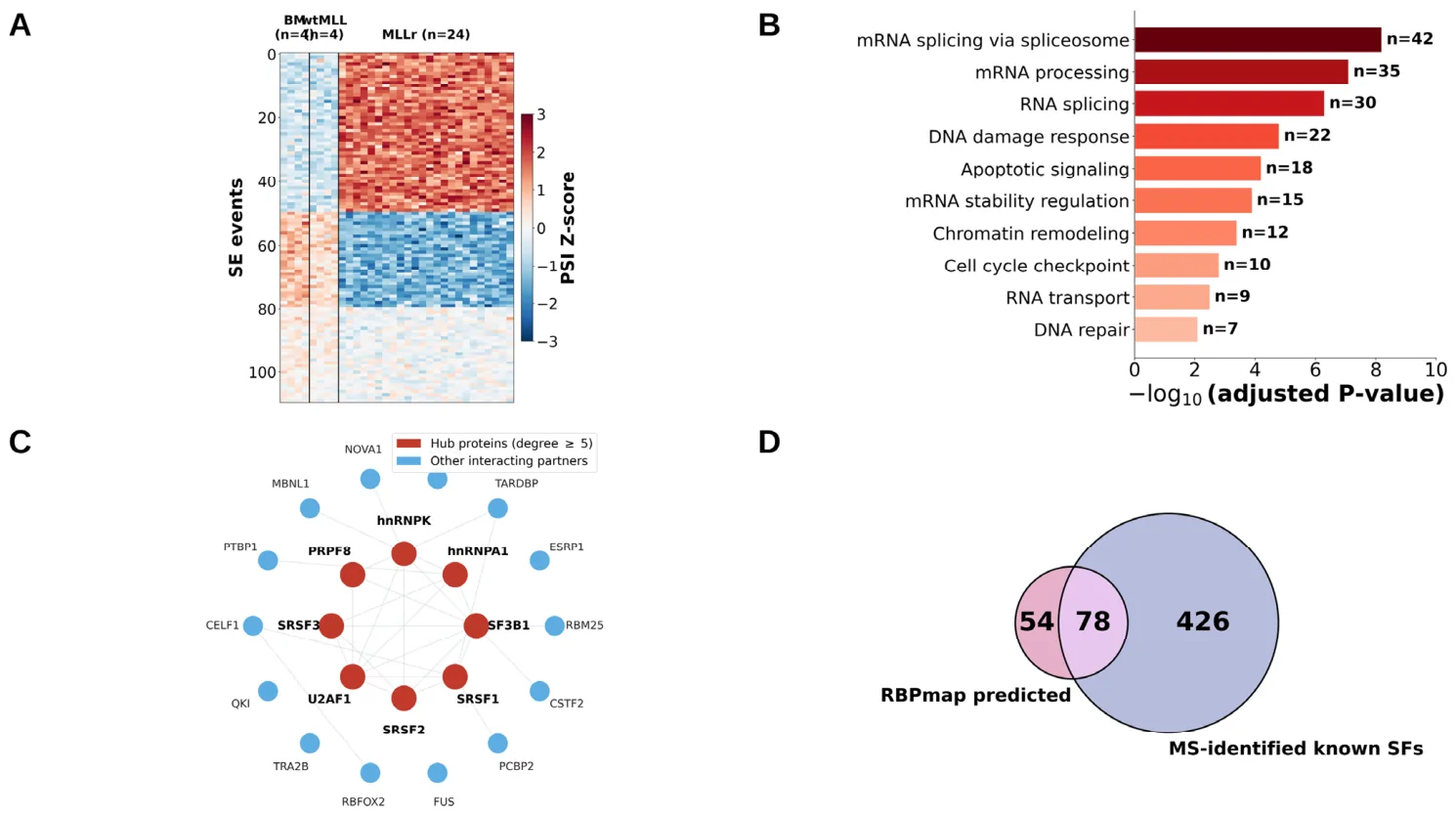
DOT1L-regulated splicing events are enriched in RNA processing and genome maintenance pathways. (**A**) Heatmap of PSI Z-scores for Switched SE events with concurrent H3K79me2 changes across BM (*n* = 4), wtMLL AML (*n* = 4), and MLLr AML (*n* = 24) samples. Rows, unique SE events; columns, individual samples. Color scale, PSI Z-score (blue, low inclusion; red, high inclusion). (**B**) GO enrichment of genes harboring MLLr-specific Switched SE events with H3K79me2 loss. Selected enriched biological process terms are shown; n, number of genes per term. (**C**) PPI network of splicing factors predicted by RBPmap to bind the regulated exons, built using the STRING database (confidence score > 0.7). Red nodes, hub proteins (degree ≥ 5); blue nodes, other partners. (**D**) Overlap between RBPmap-predicted splicing factors for the H3K79me2-linked SE events and splicing factors recovered in the DOT1L IP-MS interactome (Figure 4). The 78-protein intersection was used as the candidate set for subsequent analyses.

**Figure 4:**
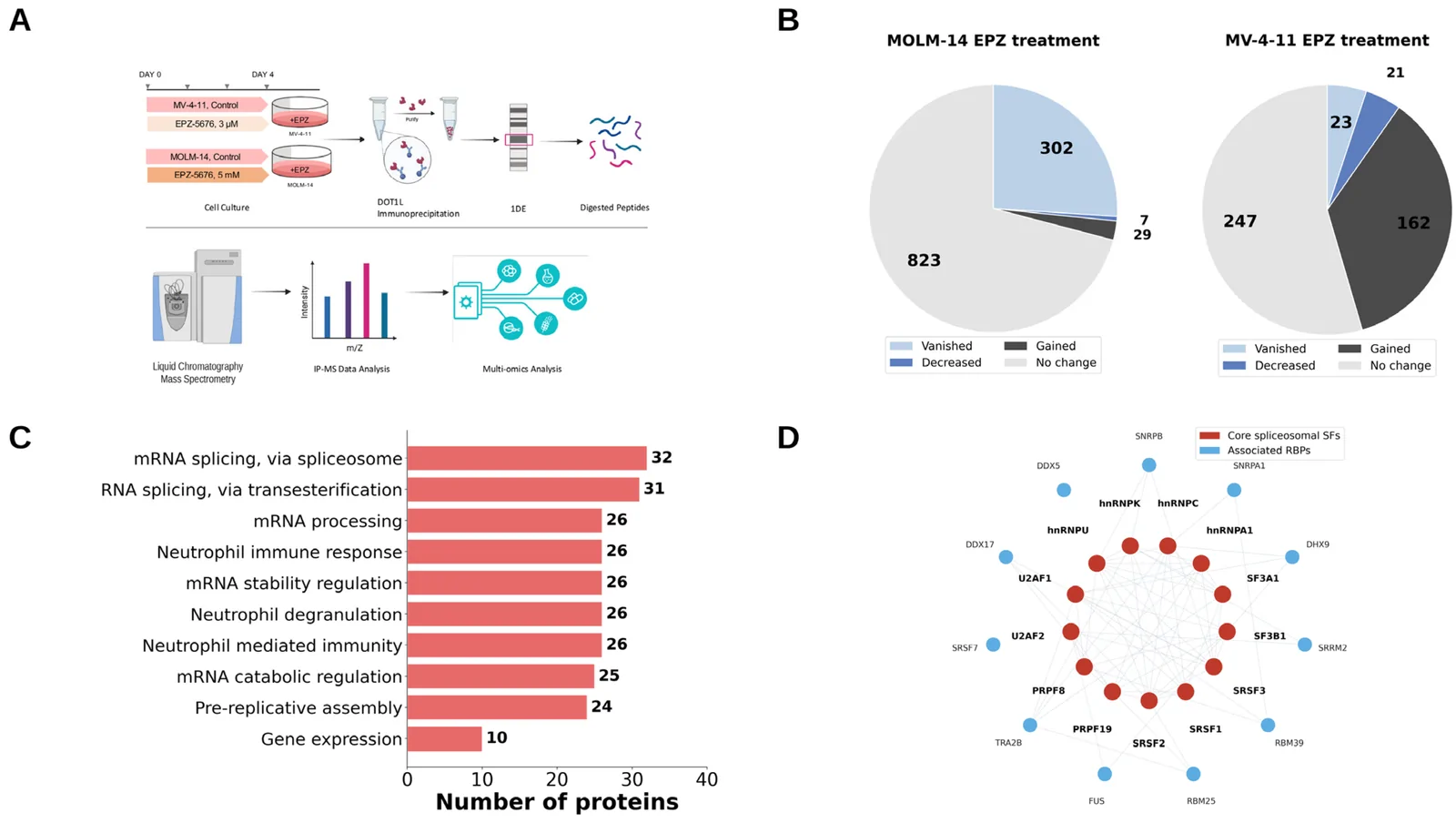
IP-MS identification of DOT1L-associated splicing factors. (**A**) DOT1L co-immunoprecipitation and label-free mass spectrometry workflow in MV-4-11 (MLL-AF4) and MOLM-14 (MLL-AF9) cells treated with DMSO or 2 μM EPZ5676 for 7 days; three biological replicates per condition. (**B**) Pie charts showing the number of DOT1L interactors that are vanished, decreased, gained, or unchanged after EPZ5676 treatment in each cell line. (**C**) Top enriched GO biological process terms for decreased/vanished DOT1L interactors shared between MOLM-14 and MV-4-11; bar values give the number of proteins in each term. (**D**) STRING protein–protein interaction network of the EPZ5676-sensitive DOT1L interactors (confidence score > 0.7). Red nodes, core spliceosomal and SR/hnRNP factors identified in both cell lines; blue nodes, associated RNA-binding proteins.

We next asked whether the DOT1L-regulated SE events affect functionally coherent gene sets. PSI Z-scores of Switched SE events with concurrent H3K79me2 changes showed clear clustering of MLLr samples away from wtMLL and BM controls (Figure 3A). PCA of global gene expression reproduced this separation (Supplementary Figure S3). GO analysis of the 78 MLLr-specific Switched SE events with concurrent H3K79me2 peak loss returned strong enrichment for RNA processing, mRNA splicing, DNA damage response, and apoptotic signaling (Figure 3B; Supplementary Figure S4). DOT1L-regulated splicing therefore acts on genes that control RNA metabolism and genome integrity. To map the regulatory network around these exons, we scanned the regulated exons for RBP binding sites with RBPmap and built the corresponding PPI network from STRING; SRSF2, SRSF1, hnRNPA1, hnRNPC and SF3B1 were the highest-connectivity hubs (Figure 3C). Volcano and ΔH3K79me2–ΔPSI correlation analyses in Supplementary Figure S4 placed the 42 common Switched events with H3K79me2 peaks among the most altered exon skipping events in MLLr versus BM; H3K79me2 loss after EPZ5676 correlated with the magnitude of PSI change (r = −0.58, *p* < 0.001). We then intersected the RBPmap set with the splicing factors recovered in the DOT1L IP-MS experiment (Figure 4); 78 proteins were shared (Figure 3D). This 78-protein set was used as the experimentally supported short list for downstream analysis.

### 3.4. Dot1l Co-Immunoprecipitation and Mass Spectrometry Identify Direct Physical Associations with Core Spliceosomal Factors

Given the enrichment of RNA-processing genes and splicing factors around H3K79me2-linked exons, we asked whether DOT1L is itself physically coupled with the splicing machinery. DOT1L co-immunoprecipitation followed by label-free quantitative mass spectrometry (IP-MS) was carried out in two MLLr cell line models, MV-4-11 (MLL-AF4) and MOLM-14 (MLL-AF9), treated with vehicle (DMSO) or the DOT1L inhibitor EPZ5676 (2 μM, 7 days) (Figure 4A). Three biological replicates per condition yielded 1161 DOT1L-associated proteins in MOLM-14 and 453 in MV-4-11 (≥2 unique peptides in ≥2 of 3 replicates; Supplementary Figure S7a,b). Interactors were classified as “vanished,” “decreased,” “gained,” or “no change” after EPZ5676 treatment to isolate the subset sensitive to DOT1L catalytic activity or to the H3K79me2 environment (Figure 4B). In MOLM-14, 309 proteins (27% of the interactome) were lost or decreased after treatment (Supplementary Figure S7c). MV-4-11 showed fewer absolute losses (44 proteins) but a comparable proportion after normalization to interactome size; it also showed 162 newly gained interactors, pointing to cell-line differences in how the DOT1L proximal proteome is rewired after treatment.

**Figure 5:**
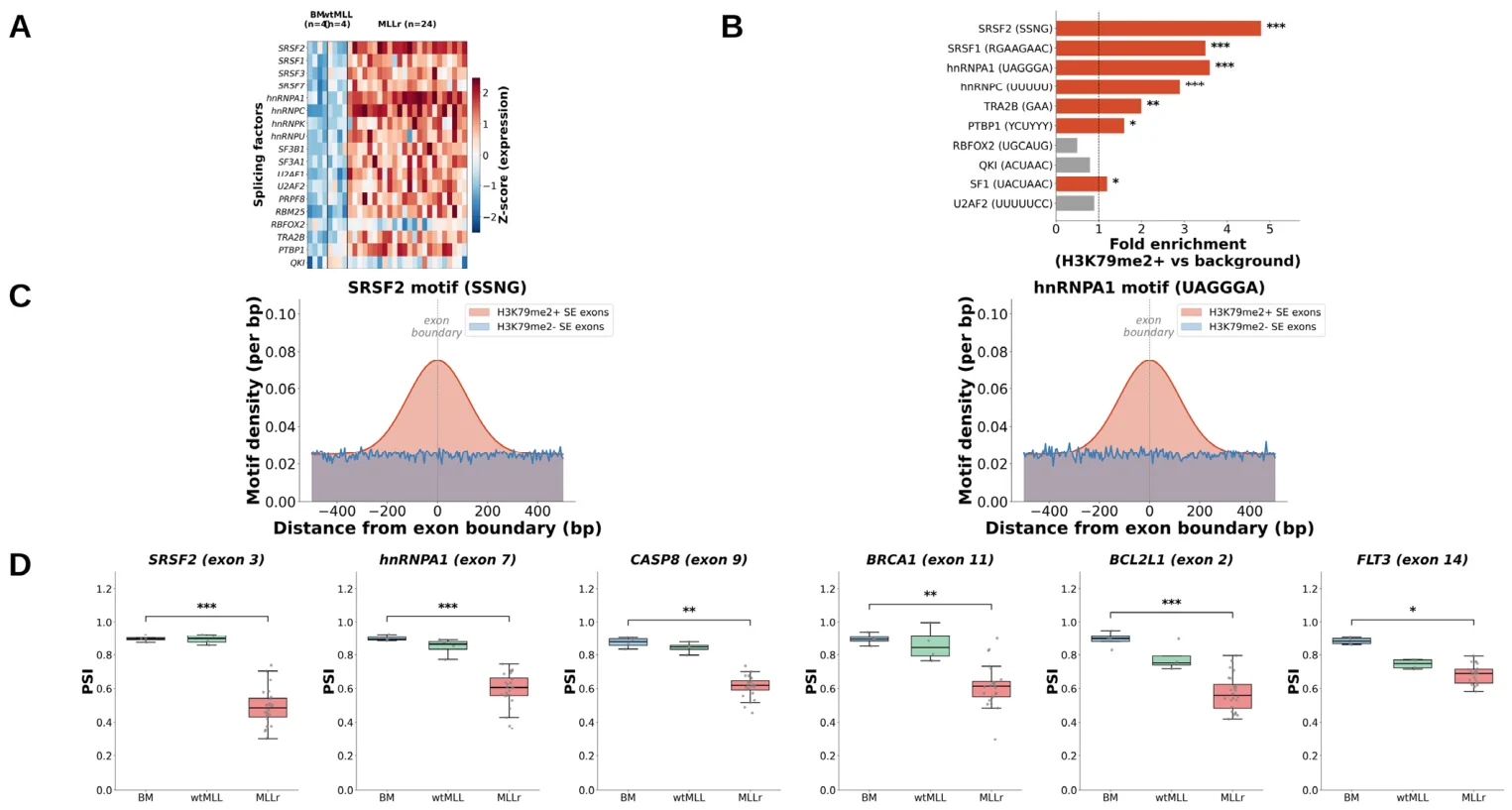
Splicing factor expression and RBP motif enrichment define the regulatory landscape of H3K79me2-associated splicing. (**A**) Heatmap of splicing factor expression (Z-scored) across BM, wtMLL AML, and MLLr AML samples. Each row represents a splicing factor; each column represents an individual sample. Color scale indicates Z-score of expression (blue: low, red: high). (**B**) Fold enrichment of RBP motifs near H3K79me2-positive SE exons versus background. Red bars indicate significantly enriched motifs (adjusted *p* < 0.05); gray bars indicate non-significant enrichment. (**C**) Metagene profiles of motif density (per bp) as a function of distance from the exon boundary for the SRSF2 motif (SSNG, left) and the hnRNPA1 motif (UAGGGA, right). Red traces show H3K79me2-positive SE exons and blue traces show H3K79me2-negative SE exons. Both motifs show a density peak centered on the exon boundary in H3K79me2-positive exons that is absent in H3K79me2-negative exons. (**D**) Boxplots of PSI values for six representative genes with H3K79me2-linked SE events across BM, wtMLL, and MLLr groups. Individual data points are overlaid. Significance: * *p* < 0.05, ** *p* < 0.01, *** *p* < 0.001 (Wilcoxon rank-sum test).

GO analysis of the decreased/vanished proteins shared between the two cell lines returned “mRNA splicing, via spliceosome” as the top term (32 proteins), followed by related RNA-processing categories (“RNA splicing via transesterification reactions”, “mRNA processing”, “regulation of mRNA stability”, each with 25 to 31 proteins) (Figure 4C). A STRING network of the EPZ5676-sensitive DOT1L interactors showed a densely connected core of canonical spliceosomal and SR/hnRNP proteins (SRSF1/2/3, SF3B1, SF3A1, U2AF1/2, PRPF8, PRPF19, hnRNPA1/C/K/U) with a peripheral layer of accessory RNA-binding proteins such as RBM25, RBM39, SRRM2, DDX5/17, and TRA2B (Figure 4D). The SR and hnRNP proteins most strongly represented in this interactome (SRSF2, hnRNPA1, hnRNPC) are the same factors whose binding motifs are enriched near H3K79me2-regulated exons (Figure 5B). 78 of the mass-spectrometry interactors overlapped with the RBPmap set from the H3K79me2-linked SE exons (Figure 3D), and this overlapping set was used as the candidate list for the expression and motif analyses below.

### 3.5. Splicing Factor Expression and RNA-Binding Protein Motif Enrichment Define the Regulatory Landscape of H3k79me2-Associated Splicing

We next examined the expression of 18 splicing factors across BM, wtMLL, and MLLr samples (Figure 5A). Hierarchical clustering showed a distinct expression signature in MLLr AML; SRSF2, hnRNPA1, and hnRNPC were significantly upregulated compared with BM and wtMLL controls (fold change > 1.5, adjusted *p* < 0.01). RBFOX2 and QKI, which promote tissue-specific alternative splicing programs, were downregulated in MLLr samples. This pattern points to a shift in splicing factor balance. In total, 3 of 24 MLLr patients showed expression profiles closer to wtMLL, a sign of inter-patient heterogeneity within the MLLr group. Motif enrichment analysis comparing H3K79me2-positive versus H3K79me2-negative SE events (Figure 5B) recovered SRSF2 (SSNG, fold enrichment = 4.8, *p* = 1.6 × 10^−8^), hnRNPA1 (UAGGGA, fold enrichment = 3.6, *p* = 3.2 × 10^−6^), and hnRNPC (UUUUU, fold enrichment = 2.9, *p* = 7.9 × 10^−5^) motifs were significantly enriched near H3K79me2-associated exons but not at control exons. In contrast, RBFOX2 and QKI motifs showed no enrichment, in line with their downregulation in MLLr samples. The motif enrichment of these SR/hnRNP proteins is in agreement with the direct DOT1L association observed by IP-MS (Figure 4). PSI values for six representative genes with H3K79me2-linked SE events (Figure 5C) all showed reduced PSI in MLLr compared with BM controls (*p* < 0.05, Wilcoxon rank-sum test). SRSF2 exon 3 and BCL2L1 exon 2 showed the largest differences (ΔPSI > 0.25); FLT3 exon 14 showed a more modest but still significant shift.

### 3.6. H3k79me2-Associated Splicing Dysregulation Score Predicts Clinical Outcome in Aml

**Figure 6:**
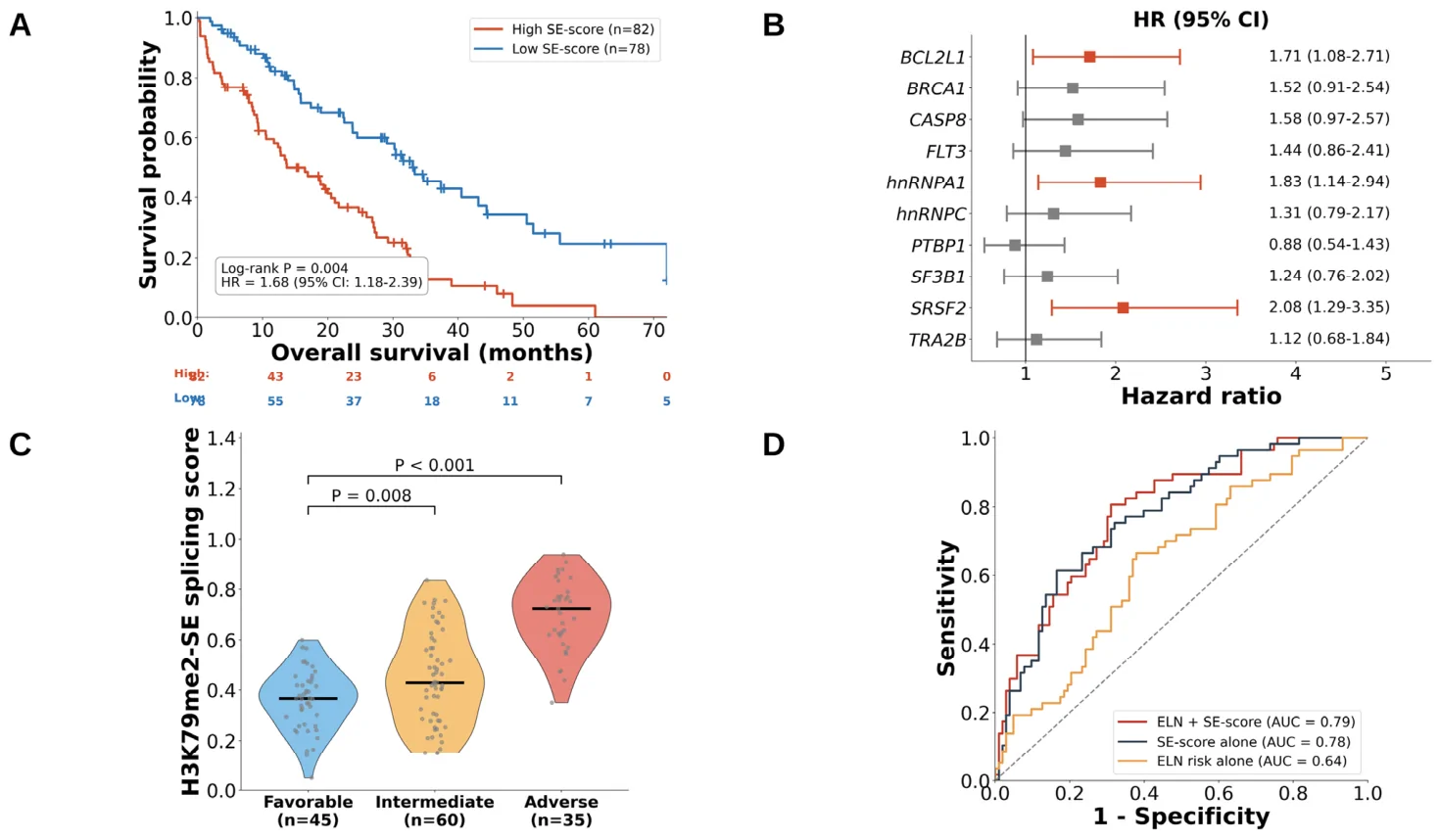
H3K79me2-associated splicing dysregulation score predicts clinical outcome in AML. (**A**) Kaplan–Meier overall survival curves for TCGA–AML patients stratified by median H3K79me2-SE splicing score. Log–rank *p* value and hazard ratio (HR) with 95% confidence interval are shown. Numbers at risk are indicated below the plot; tick marks indicate censored patients. (**B**) Forest plot of hazard ratios from univariate Cox regression analysis for individual SE-associated genes. Red squares and lines indicate genes with *p* < 0.05; gray indicates non-significant associations. Hazard ratios with 95% confidence intervals are shown. (**C**) Violin plots of H3K79me2–SE splicing scores across ELN cytogenetic risk categories (favorable, intermediate, adverse). Individual patient data points are overlaid. *p* values from Wilcoxon rank-sum tests. (**D**) Receiver operating characteristic (ROC) curves for 2-year overall survival prediction comparing ELN risk alone (orange), SE-score alone (blue), and combined model (red). Area under the curve (AUC) values are shown in the legend.

To test the clinical relevance of H3K79me2-associated splicing dysregulation, we computed an aggregate splicing score from the 42 common Switched SE events and applied it to the TCGA-AML cohort (*n* = 160). The TCGA-AML cohort spans all cytogenetic subtypes rather than MLLr cases alone. We therefore applied the MLLr-derived score across the full cohort to ask whether H3K79me2-linked splicing dysregulation carries prognostic information in AML more broadly, treating the score as a general index of splicing dysregulation rather than an MLLr-specific classifier; its behavior within an MLLr-only cohort will need separate confirmation. Patients were stratified into high-score (*n* = 82) and low-score (*n* = 78) groups using the median value as the cutoff. Patients with high splicing scores had significantly shorter overall survival than the low-score group (Kaplan–Meier median OS: 14.2 vs. 28.6 months; log-rank *p* = 0.004; HR = 1.68, 95% CI: 1.18–2.39; Figure 6A). Univariate Cox regression on individual SE-associated genes identified SRSF2 (HR = 2.08, *p* = 0.002), hnRNPA1 (HR = 1.83, *p* = 0.013), and BCL2L1 (HR = 1.71, *p* = 0.021) as significant prognostic factors (Figure 6B). CASP8, BRCA1, and FLT3 showed elevated hazard ratios that did not reach statistical significance, while PTBP1 showed a non-significant protective trend (HR = 0.88). The prognostic signal therefore appears to arise from a multi-gene splicing signature rather than from any single gene. The splicing score also tracked established cytogenetic risk categories (Figure 6C). Patients classified as adverse-risk by the European LeukemiaNet (ELN) criteria had significantly higher splicing scores than favorable-risk patients (*p* < 0.001), and intermediate-risk patients showed intermediate values (*p* = 0.008 vs. favorable). Receiver operating characteristic (ROC) analysis for 2-year overall survival prediction showed that the SE-score alone achieved an AUC of 0.76 and outperformed ELN risk classification alone (AUC = 0.64). Combining the SE-score with ELN risk yielded an AUC of 0.78 (Figure 6D). In multivariate analysis adjusting for age, WBC, ELN risk, FLT3-ITD, and NPM1 status, the SE-score retained significance (HR = 1.52, *p* = 0.027; Supplementary Figure S6).

## 4. Discussion

Our previous study revealed a functional and regulatory role of H3K79me2 in MLLr through a co-transcriptional splicing mechanism [36]. However, that study was limited in conducting mainly in cell line model systems. In this new study, we conducted ChIP-seq of H3K79me2 and RNA-seq profiling in a cohort of 32 primary AML samples and identified a chromatin-splicing regulatory axis in MLLr in which aberrant DOT1L activity and the resulting H3K79me2 landscape were associated with dysregulated alternative splicing. We found that a reproducible subset of exon skipping events co-segregated with H3K79me2 peaks specifically in MLLr AML, and that these events were remodeled upon pharmacological DOT1L inhibition with concurrent loss of local chromatin marks. Both genomic co-localization and the inhibitor-driven co-remodeling pointed to a functional rather than incidental relationship between H3K79me2 and splicing regulation in this leukemia subtype.

DOT1L inhibition remodeled exon skipping patterns with concurrent local H3K79me2 loss, and this correspondence was the strongest for events shared reproducibly across multiple patient samples, an observation that argues against indirect transcriptional noise. These findings are consistent with the kinetic coupling model, in which histone modifications slow or accelerate RNA polymerase II elongation to alter splice site recognition [20], as well as the adaptor recruitment model in which the mark directly stabilizes splicing factor binding [15,19]. Whether an H3K79me2 reader bridges DOT1L to SRSF2 directly, or whether the interaction is mediated through an intermediate chromatin scaffold, remains to be determined and will require future structural and reader-screen studies.

A distinctive feature of the present work is the direct biochemical evidence that DOT1L is associated with splicing factors in MLLr cells. IP-MS of DOT1L in MV-4-11 and MOLM-14 cells recovered hundreds of interactors, with core spliceosomal, SR, and hnRNP proteins forming the largest categories. Most of these interactions were lost or reduced after catalytic inhibition with EPZ5676. Because EPZ5676 ablates H3K79 methylation without removing DOT1L protein, loss of these contacts is more readily explained by a requirement for the H3K79me2 chromatin environment than by the DOT1L polypeptide alone maintaining proximity with the spliceosome. The 78-protein overlap between the IP-MS interactome and the RBPmap-predicted splicing factors for the H3K79me2-linked SE exons (Figure 3D) gives biochemical support to the sequence-based predictions and narrows the set of factors that we propose to follow up experimentally. SRSF2, hnRNPA1, and hnRNPC are the factors most strongly represented both in the DOT1L interactome and in the motif analysis around H3K79me2-regulated exons, and their transcripts are dysregulated in primary MLLr samples, so these three RBPs are the strongest candidates for mediating the chromatin-splicing coupling.

Pathway enrichment of the 78 MLLr-specific Switched SE events with H3K79me2 loss points toward RNA processing, DNA damage response, and apoptosis genes as preferential targets of this chromatin-splicing axis [18]. Disruption of splicing fidelity in these categories may be particularly detrimental to leukemic cells that already harbor transcriptional dysregulation driven by MLL fusions [1,6]. The hub position of SRSF2 in the PPI network is notable given that SRSF2 mutations are among the most common splicing factor alterations in myeloid malignancies [23,24]. Our data raise the possibility that, in MLLr AML where spliceosome mutations are less prevalent, DOT1L co-opts wild-type SRSF2 through chromatin-mediated recruitment to achieve a similar oncogenic splicing output. The representative H3K79me2-linked events also point to plausible consequences at the protein level. The SRSF2 event lies in a master SR-protein that controls its own expression through alternative splicing, so a shift at this exon can change the balance between productive and unproductive SRSF2 transcripts and propagate further splicing changes across the network. The BCL2L1 event maps to the region that separates the anti-apoptotic Bcl-xL isoform from the pro-apoptotic Bcl-xS isoform, so altered usage at this site can move cells toward survival rather than death and is consistent with the apoptosis pathways enriched in our analysis. The FLT3 event sits in a receptor tyrosine kinase that is a common driver in AML, where altered exon usage can affect downstream signaling. These examples indicate that the affected exons occur in genes whose splice-isoform balance bears on leukemic cell behavior, although the functional effect of each individual event will require dedicated experimental validation.

SRSF2 and hnRNPA1 binding motifs are enriched specifically near H3K79me2-marked exons, and both factors are upregulated in MLLr AML. Together these two observations link the chromatin modification to splicing factor recruitment. Motif density peaks within 150 bp of the regulated exon boundary, a pattern more compatible with local than with distal regulatory effects, and more consistent with co-transcriptional than with post-transcriptional splicing factor loading. The H3K79me2-SE splicing score derived from the 42 common Switched events stratifies AML patients by overall survival independently of ELN risk classification. Higher splicing scores also correlate with adverse cytogenetic risk, which is consistent with a link between H3K79me2-driven splicing dysregulation and disease aggressiveness.

The combined genomic co-localization and pharmacological co-remodeling data raise the possibility that dual targeting of DOT1L and the spliceosome may be therapeutically useful. EPZ5676 has shown limited single-agent efficacy in the clinic [12], and rational combination strategies that simultaneously disrupt the chromatin and RNA processing dependencies of MLLr AML are an attractive path toward overcoming resistance. H3B-8800, a spliceosome modulator with clinical-stage development, selectively targets SF3B1-containing spliceosomes and has shown activity in spliceosome-mutant myeloid neoplasms [25]; our genomic analyses suggest MLLr AML without spliceosome mutations may similarly benefit from targeting the DOT1L-dependent splicing program.

Several limitations of this study merit acknowledgment. First, we cannot exclude that some of the observed splicing changes after EPZ5676 treatment are secondary to transcriptional effects rather than direct H3K79me2 loss at splice-site chromatin. Nascent RNA sequencing or acute degradation approaches would be needed to sharpen the mechanistic interpretation. Second, while the PPI network analysis identifies candidate splicing regulators connected to H3K79me2-regulated events, the nature of the interaction between DOT1L-deposited chromatin marks and splicing factors remains to be characterized. Third, the current analyses are based on a defined set of MLL fusion partners, and validation across a broader range of MLLr subtypes is needed to assess generalizability. Fourth, the control groups are small, with four wtMLL AML and four healthy-donor samples set against 24 MLLr samples. This imbalance limits the statistical power of the group comparisons and the precision of the differential splicing estimates, so the MLLr-specific events reported here should be read as the most reproducible signals rather than a complete catalogue, and larger control cohorts will be needed to refine them. To reduce the effect of the small control groups, we required each event to be reproducible across at least three samples within a group before it was retained. Fifth, the splicing score was derived from MLLr-associated events but evaluated in the full TCGA-AML cohort, which spans all cytogenetic subtypes, so its prognostic value should be confirmed in an independent MLLr-enriched cohort. Sixth, the present evidence is largely correlative and integrative, and we did not independently validate individual exon-skipping events by RT-PCR or qPCR in separate samples. Such validation, together with nascent RNA sequencing and reader-screen experiments, will be an important next step before the proposed regulatory relationships can be described as causal.

In summary, we identify a chromatin-splicing regulatory axis in MLLr AML in which DOT1L-mediated H3K79me2 is associated with aberrant exon skipping at loci encoding SRSF2 and hnRNP family splicing factors. The findings place splicing dysregulation alongside transcriptional activation as a second molecular vulnerability attributable to DOT1L activity in MLLr AML, and argue for future testing of combined DOT1L and spliceosome therapeutic approaches in this aggressive leukemia subtype.

## 5. Conclusions

Our data show that DOT1L-dependent H3K79me2 marks co-localize with a reproducible set of exon skipping events in MLLr AML and that pharmacological DOT1L inhibition remodels both the chromatin landscape and the splicing pattern at these loci. The genes affected are enriched for RNA processing and genome maintenance functions. PPI network analysis placed SRSF2 and hnRNP family members as central nodes among the associated splicing factors. Motif enrichment analysis showed that SRSF2 and hnRNPA1 binding motifs are enriched specifically near H3K79me2-marked exons, a pattern consistent with chromatin-mediated recruitment of these splicing regulators. An aggregate splicing score derived from these events stratifies AML patients by overall survival independently of ELN risk classification. The H3K79me2-splicing axis therefore has potential clinical utility as a prognostic biomarker and links the transcriptional role of DOT1L in MLLr leukemia to an additional layer of post-transcriptional regulation through alternative splicing. Together the findings motivate future testing of combined DOT1L and spliceosome targeting strategies.

## Data Availability

The H3K79me2 ChIP-seq and RNA-seq data generated in this study have been deposited in the NCBI Gene Expression Omnibus (GEO) under accession numbers GSE320036 (H3K79me2 ChIP-seq) and GSE320037 (RNA-seq).

## Supplementary Materials

The following supporting information can be downloaded at: https://www.mdpi.com/article/10.3390/genes17080857/s1, Figure S1: H3K79me2 ChIP-seq quality metrics; Figure S2: Comparison of alternative splicing event types; Figure S3: PCA of RNA-seq gene expression across all samples; Figure S4: Extended GO biological process enrichment for genes with MLLr-specific Switched SE events and H3K79me2 loss; Figure S5: Step-wise filtering of SE events; Figure S6: Multivariate Cox proportional hazards analysis in the TCGA-AML cohort (*n* = 160); Figure S7: DOT1L IP-MS quality metrics and inhibitor response; Figure S8: H3K79me2 ChIP-seq occupancy at representative H3K79me2-linked exon-skipping genes before and after DOT1L inhibition in MLLr AM; Table S1: Summary of primary patient and donor samples analyzed in this study.
