# Supplementary material for: Genome-Wide Identification of a Chromatin-Splicing Regulatory Axis Driven by DOT1L in MLL-Rearranged Acute Myeloid Leukemia
Source: Genes (Basel). 2026 Jul 24;17(8):857. doi: 10.3390/genes17080857 (PMC13512208; doi:10.3390/genes17080857)
Supplement: Supplementary file 1 [file genes-17-00857-s001.zip › genes-4357824-supplementary.pdf]

Supplementary Materials

A chromatin-splicing regulatory axis driven by DOT1L defines transcriptomic vulnerability in MLL-rearranged acute myeloid leukemia

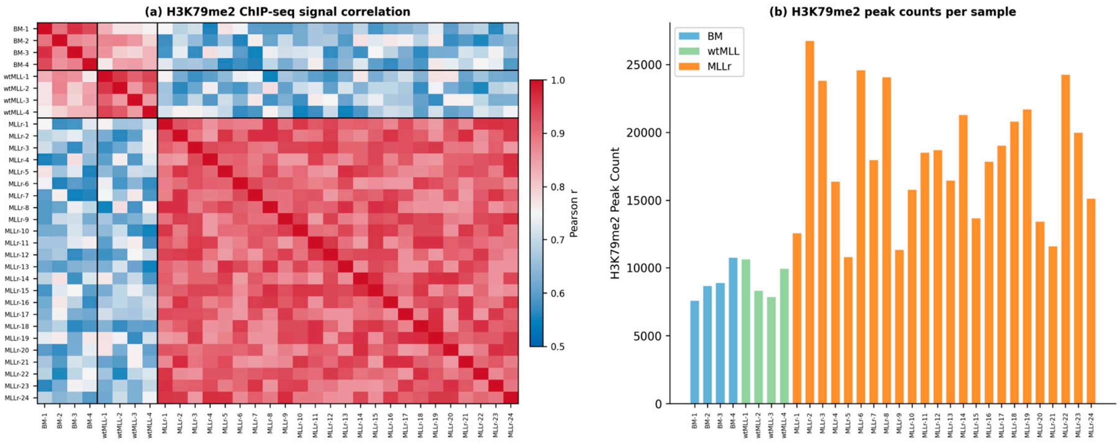

Figure S1. H3K79me2 ChIP-seq quality metrics. (a) Pearson correlation of genome-wide H3K79me2 ChIP-seq signal between all 32 samples. Black lines separate BM, wtMLL, and MLLr groups. (b) Number of H3K79me2 peaks called per sample (MACS2, q < 0.05, fold enrichment > 2).

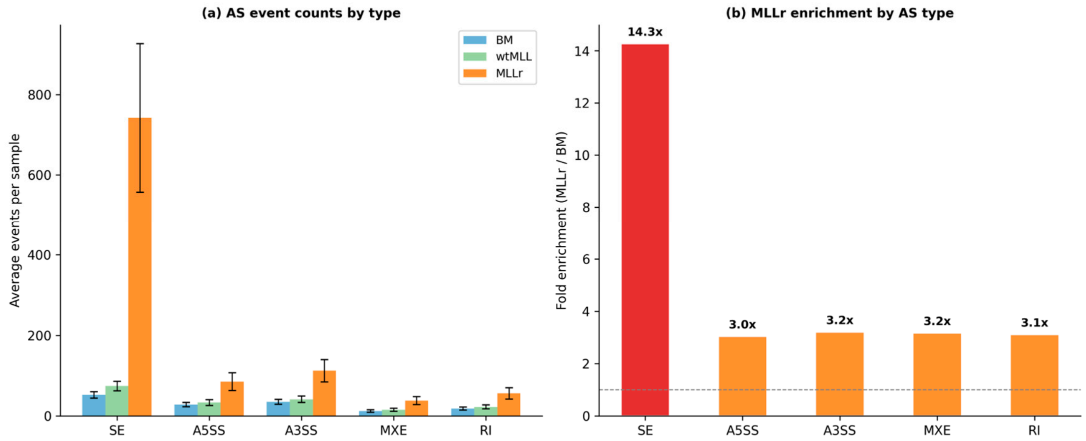

Figure S2. Comparison of alternative splicing event types. (a) Mean number of events per sample for each splicing type (SE, A5SS, A3SS, MXE, RI) in BM, wtMLL, and MLLr groups. Error bars, s.d. (b) Fold change of event counts in MLLr relative to BM for each type.

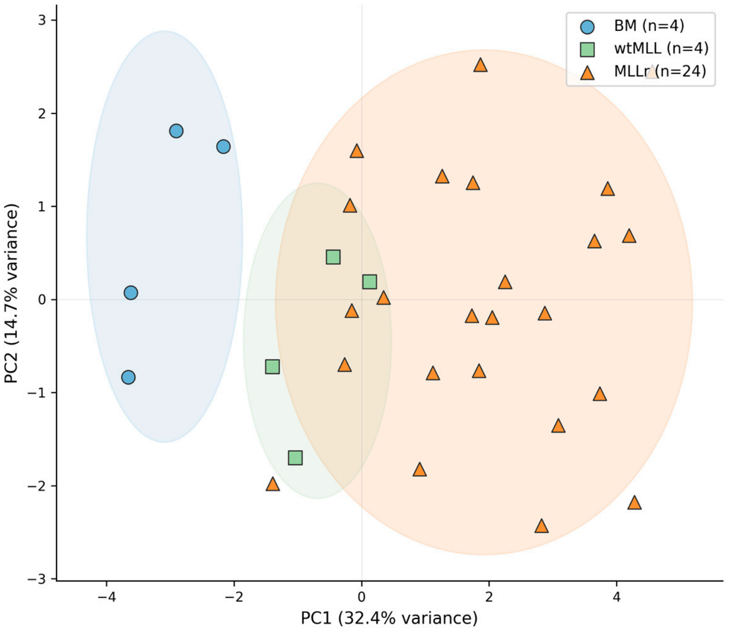

Figure S3. PCA of RNA-seq gene expression across all samples. Variance-stabilized expression values were used as input. Ellipses denote 95% confidence regions. Percentage of variance explained by each component is shown on the axes.

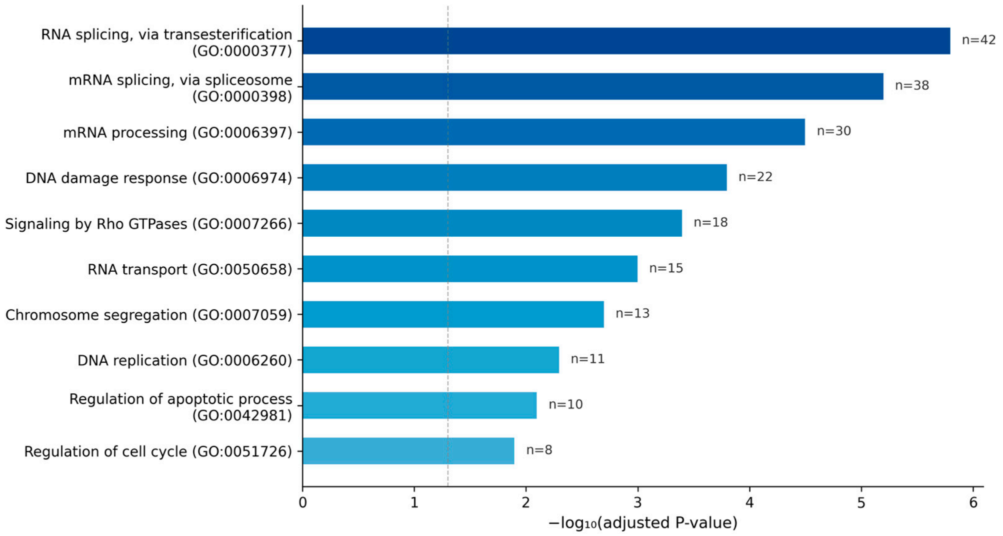

Figure S4. Extended GO biological process enrichment for genes with MLLr-specific Switched SE events and H3K79me2 loss. The top 10 terms ranked by adjusted P-value are shown. n, number of input genes annotated to each term. Dashed line, P = 0.05.

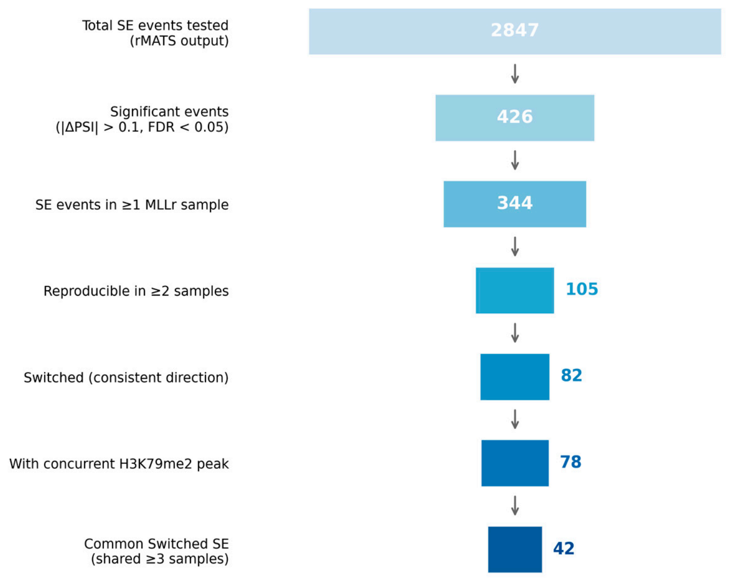

Figure S5. Step-wise filtering of SE events. Starting from 2,847 rMATS-called SE events, successive filters for statistical significance, reproducibility, directional consistency, and H3K79me2 peak overlap yielded 42 common Switched events used in clinical analyses.

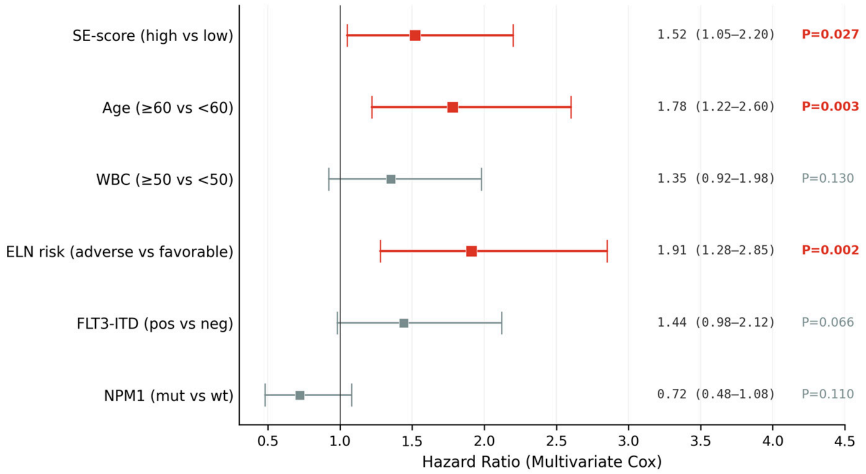

Figure S6. Multivariate Cox proportional hazards analysis in the TCGA-AML cohort (n = 160). Covariates include the H3K79me2-SE splicing score (dichotomized at median), age (≥60 vs. <60), WBC count (≥50 vs. <50 × 10^9/L), ELN risk category, FLT3-ITD status, and NPM1 mutation status. Red, $P < 0.05$; gray, not significant. Error bars, 95% CI.

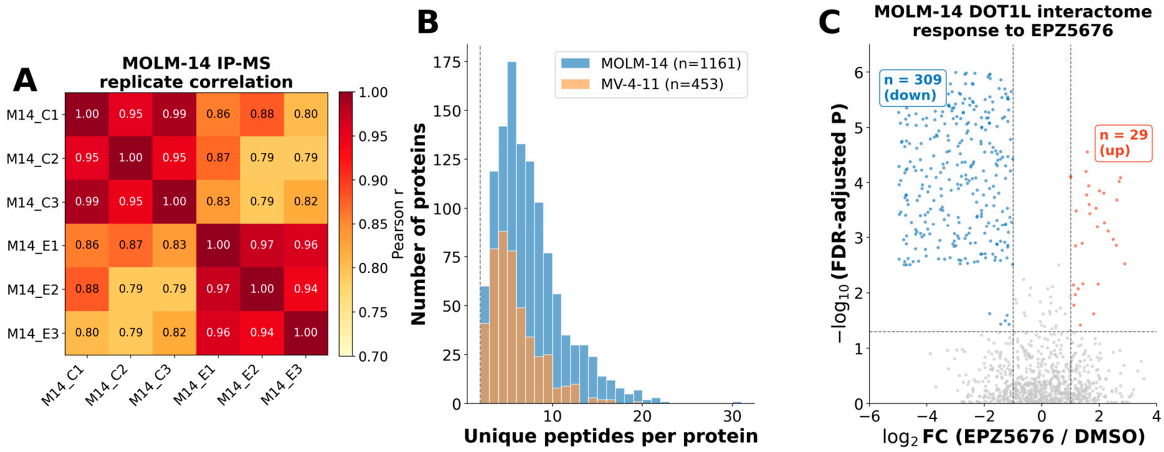

Figure S7. DOT1L IP-MS quality metrics and inhibitor response. (a) Pearson correlation matrix of normalized peptide intensities for the three control (M14_C1–C3) and three EPZ5676-treated (M14_E1–E3) MOLM-14 IP-MS replicates. Within-condition correlations exceed 0.94. (b) Distribution of unique peptide counts per protein for MOLM-14 (n = 1,161) and MV-4-11 (n = 453) interactomes. Dashed line, retention threshold (≥ 2 unique peptides). (c) Volcano plot of MOLM-14 DOT1L interactor abundance after EPZ5676 treatment. Blue, decreased or vanished interactors ($\log_2$FC ≤ −1, FDR-adjusted P < 0.05; n = 309); red, gained interactors ($\log_2$FC ≥ 1, FDR-adjusted P < 0.05; n = 29); gray, no significant change. Dashed lines, significance thresholds.

Supplementary Table S1. Summary of primary patient and donor samples analyzed in this study.

Primary bone marrow and peripheral blood samples profiled by H3K79me2 ChIP-seq and RNA-seq, comprising 4 healthy bone marrow donors, 4 wild-type MLL (wtMLL) AML patients, and 24 MLL-rearranged (MLLr) AML patients. Each MLLr patient was profiled before (None) and after DOT1L inhibition with EPZ5676, giving 48 MLLr samples and 56 samples in total. All data are deposited in the Gene Expression Omnibus under accessions GSE320036 (ChIP-seq) and GSE320037 (RNA-seq).

No.	Group	Sample ID	Tissue	DOT1L inhibitor	GEO accession
1	Healthy bone marrow donor	BM_20070422	Bone marrow	None	GSM9532574
2	Healthy bone marrow donor	BM_200804711	Bone marrow	None	GSM9532573
3	Healthy bone marrow donor	BM_ND16600	Bone marrow	None	GSM9532575
4	Healthy bone marrow donor	BM_RIJIV1	Bone marrow	None	GSM9532576
5	wtMLL AML	PS_78299	Peripheral blood	None	GSM9532579
6	wtMLL AML	PS_88351	Peripheral blood	None	GSM9532578
7	wtMLL AML	PS_743835_7	Peripheral blood	None	GSM9532580
8	wtMLL AML	PS_754422_2	Peripheral blood	None	GSM9532577
9	MLLr AML	L12	Peripheral blood	None	GSM9532603
10	MLLr AML	L14	Peripheral blood	None	GSM9532604
11	MLLr AML	MR1	Peripheral blood	None	GSM9532581
12	MLLr AML	MR2	Peripheral blood	None	GSM9532582
13	MLLr AML	MR3	Peripheral blood	None	GSM9532583
14	MLLr AML	MR4	Peripheral blood	None	GSM9532584
15	MLLr AML	MR5	Peripheral blood	None	GSM9532585
16	MLLr AML	MR6	Peripheral blood	None	GSM9532586
17	MLLr AML	MR7	Peripheral blood	None	GSM9532587
18	MLLr AML	MR8	Peripheral blood	None	GSM9532588
19	MLLr AML	MR9	Peripheral blood	None	GSM9532589
20	MLLr AML	MR10	Peripheral blood	None	GSM9532590
21	MLLr AML	Q5	Peripheral blood	None	GSM9532591
22	MLLr AML	Q6	Peripheral blood	None	GSM9532592
23	MLLr AML	Q9	Peripheral blood	None	GSM9532593
24	MLLr AML	Q10	Peripheral blood	None	GSM9532594
25	MLLr AML	Q11	Peripheral blood	None	GSM9532595
26	MLLr AML	Q12	Peripheral blood	None	GSM9532596
27	MLLr AML	Q13	Peripheral blood	None	GSM9532597
28	MLLr AML	Q14	Peripheral blood	None	GSM9532598
29	MLLr AML	Q15	Peripheral blood	None	GSM9532599
30	MLLr AML	Q16	Peripheral blood	None	GSM9532600
31	MLLr AML	Q17	Peripheral blood	None	GSM9532601
32	MLLr AML	Q18	Peripheral blood	None	GSM9532602
33	MLLr AML	L13	Peripheral blood	EPZ5676 (pinometostat)	GSM9532627
34	MLLr AML	L15	Peripheral blood	EPZ5676 (pinometostat)	GSM9532628
35	MLLr AML	T1	Peripheral blood	EPZ5676 (pinometostat)	GSM9532621
36	MLLr AML	T2	Peripheral blood	EPZ5676 (pinometostat)	GSM9532626
37	MLLr AML	T3	Peripheral blood	EPZ5676 (pinometostat)	GSM9532625
38	MLLr AML	T4	Peripheral blood	EPZ5676 (pinometostat)	GSM9532624
39	MLLr AML	T5	Peripheral blood	EPZ5676 (pinometostat)	GSM9532616
40	MLLr AML	T6	Peripheral blood	EPZ5676 (pinometostat)	GSM9532620
41	MLLr AML	T7	Peripheral blood	EPZ5676 (pinometostat)	GSM9532615
42	MLLr AML	T8	Peripheral blood	EPZ5676 (pinometostat)	GSM9532617
43	MLLr AML	T9	Peripheral blood	EPZ5676 (pinometostat)	GSM9532618
44	MLLr AML	T10	Peripheral blood	EPZ5676 (pinometostat)	GSM9532623
45	MLLr AML	T11	Peripheral blood	EPZ5676 (pinometostat)	GSM9532622
46	MLLr AML	T12	Peripheral blood	EPZ5676 (pinometostat)	GSM9532619
47	MLLr AML	TMR1	Peripheral blood	EPZ5676 (pinometostat)	GSM9532605
48	MLLr AML	TMR2	Peripheral blood	EPZ5676 (pinometostat)	GSM9532606
49	MLLr AML	TMR3	Peripheral blood	EPZ5676 (pinometostat)	GSM9532607
50	MLLr AML	TMR4	Peripheral blood	EPZ5676 (pinometostat)	GSM9532608
51	MLLr AML	TMR5	Peripheral blood	EPZ5676 (pinometostat)	GSM9532609
52	MLLr AML	TMR6	Peripheral blood	EPZ5676 (pinometostat)	GSM9532610
53	MLLr AML	TMR7	Peripheral blood	EPZ5676 (pinometostat)	GSM9532611
54	MLLr AML	TMR8	Peripheral blood	EPZ5676 (pinometostat)	GSM9532612
55	MLLr AML	TMR9	Peripheral blood	EPZ5676 (pinometostat)	GSM9532613
56	MLLr AML	TMR10	Peripheral blood	EPZ5676 (pinometostat)	GSM9532614

MLL rearrangement status was determined by fluorescence in situ hybridization (FISH) and/or reverse transcription PCR (RT-PCR). Patient-level clinical annotation (age, sex, white blood cell count, and individual MLL fusion partner) is not included because the primary samples are deidentified under the governing institutional review board approvals.

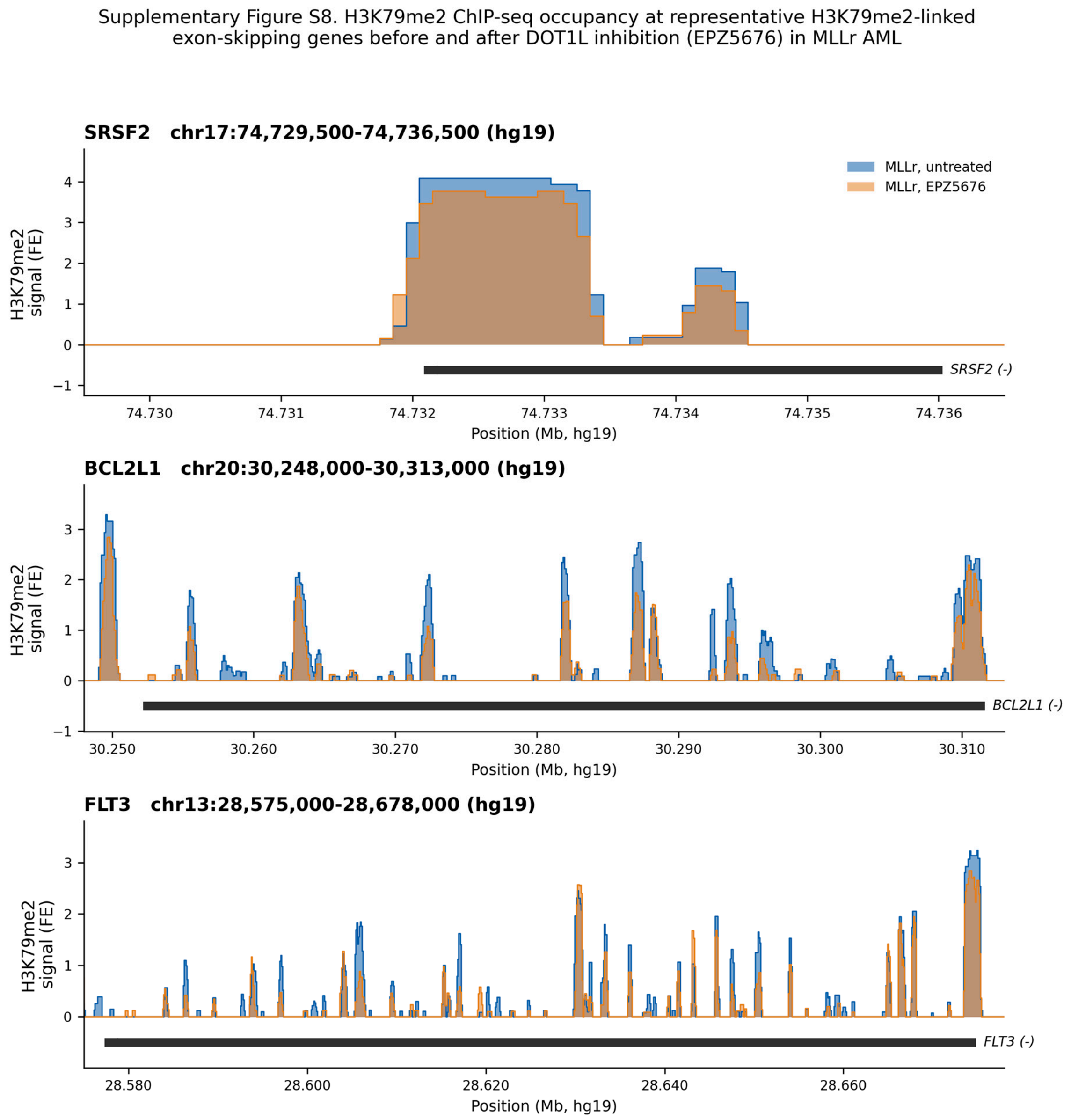

Supplementary Figure S8. H3K79me2 ChIP-seq occupancy at representative H3K79me2-linked exon-skipping genes before and after DOT1L inhibition in MLLr AML. Genome-browser tracks show H3K79me2 ChIP-seq signal (MACS2 broadPeak fold-enrichment) across the SRSF2 (chr17), BCL2L1 (chr20), and FLT3 (chr13) loci (hg19), averaged across untreated MLLr AML samples (blue) and EPZ5676-treated MLLr AML samples (orange). The gene body and direction of transcription are drawn below each track. At all three loci the local H3K79me2 signal is reduced after EPZ5676 treatment, consistent with the genome-wide peak loss in Figure 2d and with the exon-skipping (PSI) changes quantified in Figure 5c. ChIP-seq data are from GEO accession GSE320036 and RNA-seq data from GSE320037.
